# Epigallocatechin-3-gallate and its nanoformulation in cervical cancer therapy: the role of genes, MicroRNA and DNA methylation patterns

**DOI:** 10.1186/s12935-023-03161-9

**Published:** 2023-12-21

**Authors:** Guichun Wang, Jinyi Wang, Mohammad Reza Momeni

**Affiliations:** 1 School of Traditional Chinese Medicine, Beijing University of Chinese Medicine, Beijing, 100029 China; 2 School of Clinical Medicine, Tsinghua University, Beijing, 100084 China; 3 School of Medicine, Tehran University of Medical Sciences, Tehran, Iran

**Keywords:** Nanotechnology, Green tea, Epigallocatechin-3-gallate, Cervical cancer, DNA methylation, MicroRNA

## Abstract

Green tea, a popular and healthy nonalcoholic drink consumed globally, is abundant in natural polyphenols. One of these polyphenols is epigallocatechin-3-gallate (EGCG), which offers a range of health benefits, such as metabolic regulation, antioxidant properties, anti-inflammatory effects, and potential anticancer properties. Clinical research has shown that EGCG can inhibit cancers in the male and female reproductive systems, including ovarian, cervical, endometrial, breast, testicular, and prostate cancers. Further research on cervical cancer has revealed the crucial role of epigenetic mechanisms in the initiation and progression of this type of cancer. These include changes to the DNA, histones, and non-coding RNAs, such as microRNAs. These changes are reversible and can occur even before genetic mutations, making them a potential target for intervention therapies. One promising approach to cancer prevention and treatment is the use of specific agents (known as epi-drugs) that target the cancer epigenome or epigenetic dysregulation. Phytochemicals, a group of diverse molecules, have shown potential in modulating cancer processes through their interaction with the epigenetic machinery. Among these, green tea and its main polyphenol EGCG have been extensively studied. This review highlights the therapeutic effects of EGCG and its nanoformulations on cervical cancer. It also discusses the epigenetic events involved in cervical cancer, such as DNA methylation and microRNA dysregulation, which may be affected by EGCG.

## Introduction

Cervical cancer is the third most typical malignancy in females and the fourth deadliest disease worldwide [1]. It continues to be the second most common reason for female cancer death in underdeveloped nations [2]. About 3.7% of new cancer diagnoses and 3.2% of cancer deaths are caused by cervical cancer [1]. In US, there are around 105,890 new instances of cancer in females’ genital system, including 12,990 instances of cancer of the uterine cervix [3]. Cervical cancer accounts for 4120 of the 30,890 deaths [3]. Following uterine cervix (31.7%), vagina (20.5%), vulva (18.6%), as well as ovarian cancer (17.4%) in order of mortality rate, ovarian cancer has the highest death rate (63.9%). Breast, vaginal, and thyroid cancers are much frequent in young female under the age of 50. Due to the fact that cervical cancer is among the main contributers of mortality and morbidity in females across the world, much research has been done to develop a variety of therapies like vaccines and medications, in order to treat it at various phases. Chemotherapy, which combines certain cytotoxic drugs with anti-metabolites like DNA-interactive and methotrexate agents like cisplatin and doxorubicin, is the most popular method for controlling cervical cancer.

One of the top three non-alcoholic beverages in the world is tea. (-)-epigallocatechingallate (EGCG), (-)-epigallocatechin, (-)-epicatechingallate, and (-)-epicatechin are the four main catechins found in green tea [4]. Over 40% of green tea’s total catechins, or EGCG, are known to be beneficial for preventing cancer, diabetes, obesity, neurological diseases, stroke, and other biochemical problems [5–9]. The results of epidemiological, in vitro and in vivo investigations provide strong evidence for effectiveness of EGCG in preventing cancer [10–15], particularly in breast cancer [16, 17], liver cancer [18], and prostate cancer [19]. The advantages of EGCG in preventing cervical cancer are still debatable and unclear though.

Because of their small size, nano-sized phytochemicals (NPCs) like EGCG have piqued the curiosity of scientists. Because of its gradual rate of release into the bloodstream, EGCG has increased bioavailability and absorption. Another benefit is the fact that they are only required in trace amounts, which lowers the overall cost of therapy. As a result, using EGCG exclusively or in conjunction with established therapy regimens may significantly improve results of therapy and lengthen patients’ lives who have cervical cancer. Epigenetic modifications, in addition to genetic changes, are crucial for the neoplastic growth and progression of cervical cancer [15]. These include non-coding microRNAs, histone alterations, and DNA methylation. A brand-new family of short, 19–25 nucleotide, non-coding RNAs called miRNAs normally silences the expression of genes [20–24]. Given that early carcinogenesis epigenetic changes are reversible, reversing (correcting) these modifications with green tea catechins may be a potential approach for cancer chemoprevention and treatment [16]. Recent research reveals that green tea catechins like epigallocatechin gallate (EGCG) can alter miRNA expression and their target mRNAs in addition to acting as epigenetic modulators, hence persistently inhibiting cervical cancer.

In this regard, to help in the search for more effective and suitable treatments for individuals with cervical cancer, we made an effort to analyze the modes of action of green tea as well as EGCG when given to cervical cancer cells and patients. This review highlights the therapeutic effects of EGCG and its nanoformulations on cervical cancer. It also discusses the epigenetic events involved in cervical cancer, such as DNA methylation and microRNA dysregulation, which may be affected by EGCG.

## Grean tea and EGCG, and cervical cancer

The fourth most prevalent and the main reason for cancer-related fatalities in women, respectively, is cervical cancer [25]. It has been established that oncogenic HPV16 and HPV18 infections are the cause of cancer. Additional hazards are smoking, a record of various sexual partners, unsafe liaisons, as well as early sexual behavior [26]. HPV vaccine offers this cancer protection, although improvements in the management of the condition are still required. It has been established that green tea is useful in the therapy of cervical cancer due to its main active ingredient, EGCG, as well as its alleged anti-cancer characteristics (Table 1).

**Table 1:** Therapeutic effects of active ingredients in green tea on cervical cancer

Compound	Dose	Target	Effects	Model	Cell line	Ref
Epigallocatechin gallate (EGCG) and polyphenols E	0–50 µg/ml	HPV-E7	Inhibited immortalized cervical epithelial and cancer cell growth, apoptosis is induced by EGCG in a dose-dependent statistically	*In-vitro*	TCL1, HeLa, Me180	[27]
EGCG	250–500 µM	-	Induced cell cycle arrest and apoptosis, Inhibited cell Growth	*In-vitro*	HeLa, C33A, WI-38	[28]
EGCG	25 µM	NFκB p65, COX-2, p-Akt, p-mTOR	Inhibited growth of cervical cancer cells, inhibited cell survival and induced apoptosis	*In-vitro*	HeLa	[29]
Sinecatechins	160 to 360µM	-	Inhibited cancer cell growth, induced apoptosis, mediated by cell cycle deregulation	*In-vitro*	CaSki, SiHa, HeLa, C4-I	[30]
Green tea	-	-	A protective factor against cervical cancer	*Human*	-	[31]
Polyphenon E	800 mg	CIN1	Did not promote the clearance of persistent high-risk HPV and related CIN1	*Human*	-	[32]
EGCG	60 µg/mL	-	Cytostatic but not cytotoxic effect	*In-vitro*	HeLa	[33]
EGCG	1-100 µM	MMP-9, TIMP-1	Induced apoptosis and inhibited invasion and migration	*In-vitro*	HeLa	[34]
epigallocatechin-3-gallate	50 µg/ml	SOD, GPx	Free radical scavenging properties, protected the proliferation of cancerous cells and boosted the activity of SOD and GPx	*In-vitro, human*	HeLa	[35]
EGCG	35 µM	-	Inhibited cervical cancer cell growth through induction of apoptosis and cell cycle arrest	*In-vitro*	CaSki	[36]
EGCG	0, 25, 50 µM	-	Inhibited proliferation, induced cell cycle arrest	*In-vitro*	HeLa	[37]
EGCG	25 µM	DNMT3B	Reversed expression of various tumor-suppressor genes by inhibiting DNA methyltransferases and histone deacetylases	*In-vitro*	HeLa	[38]
EGCG	25 or 50 µM	p53	Suppressed Fused Toes Homolog protein	*In-vitro*	HeLa	[39]
EGCG	25–100 µg/ml	cyclinD1, LIMD1, RBSP3, p16, DNMT1	Inhibited cellular proliferation, Induced apoptosis	*In-vitro*	HeLa	[40]
Catechin hydrate (CH)	50–600 lg/mL	tp53, caspase-3, -8, -9	Inhibited proliferation and mediated apoptosis	*In-vitro*	SiHa	[41]
Tea extract and EGCG	0-100 µM /L	TGF-β	Suppressed TGF-β-induced EMT, decreased ROS levels	*In-vitro*	Hela, SiHa	[42]
EGCG	50–100 µM	DNMT, HDAC, HMT, H3K9	Decreased global DNA methylation	*In-vitro*	HeLa	[43]
EGCG	10 µM	-	Affected proliferation, adhesion and motility	*In-vitro*	HeLa	[44]
EGCG, tea polyphenol	0–100 mM	IGF-IR	Inhibited the proliferation, induced cell apoptosis	*In-vitro*	OMC-4, TMCC-1	[45]
EGCG	0-100 µg/ml	miR-203, miR-125b	Inhibited cell proliferation	*In-vitro*	HeLa, CaSki, C33A	[46]
EGCG	0-100 µM	-	Antiproliferative effects	*In-vitro*	HeLa and TMCC-1	[47]
EGCG	25, 50 µM	-	Inhibited Cell growth and poliferation	*In-vitro*	CaSki, HeLa	[48]
EGCG	5, 10 µg/ml	caspase-3	Increased number of apoptotic cells	*In-vitro*	PBMCs	[49]
Green tea extract, EGCG	80 µg/mL, 100 µmol/L	VEGF	Abolished cell migration	*In-vitro*	HeLa, HepG2	[50]

## Potential of grean tea and EGCG as an autophagy modulator in cervical cancer

Because the pharmacological effects of autophagy modulators aren’t tumor-selective, there is the possibility that normal tissue might develop to cancerous tissue [51, 52]. Although autophagy has been found to cause cancer cell death, green tea polyphenols’ anti-oxidative capabilities reduced stress-induced autophagy cell death in healthy ovarian granulosa cells. SOD and GSH-PX antioxidant activities along with concentrations have been increased. Beclin1, MDA and autophagic vacuoles in the cytoplasm were all reduced [53]. In healthy ovarian cells, ROS reduction caused via EGCG limited the enzyme which split LC3-I into LC3-II as well as reduced the production of LC3-II, indicating less autophagy as well as autophagosome formation [54, 55]. Environmental stress from toxins and pollutants encouraged autophagy [56]. EGCG has the ability to control autophagy and guard against chemotherapy-induced cell death in normal cells.

Green tea polyphenol treatment in cervical cancer increased autophagy and death in HPV-16-positive cells, according to Tang et al. [57], whereas Nrf2 gene knockdown prevented green tea polyphenol-mediated autophagy and apoptosis. Furthermore, Doxorubicin, a chemotherapeutic, has been used with EGCG in studies as an autophagy regulator that boosted photothermal therapy’s therapeutic effectiveness [58]. Photothermal treatment prevented apoptosis-induced cancer cell death by inducing autophagy and only hyperthermic harm to intracellular elements [59, 60]. An autophagy enhancer, on the other hand, may inhibit the spread of cancer and inflammation, hence promoting autophagy-induced cell death. Once autophagosome production and autophagic flux were triggered, p62 was downregulated whereas Beclin1 and LC3 were upregulated in malignancies. Furthermore, EGCG reduced chemo-resistance and reduced the toxins Doxorubicin generated in vivo and in vitro, leading to increased effectiveness [58, 61].

## Potential effects of grean tea and EGCG on the apoptotic cascade

**Fig. 1:**
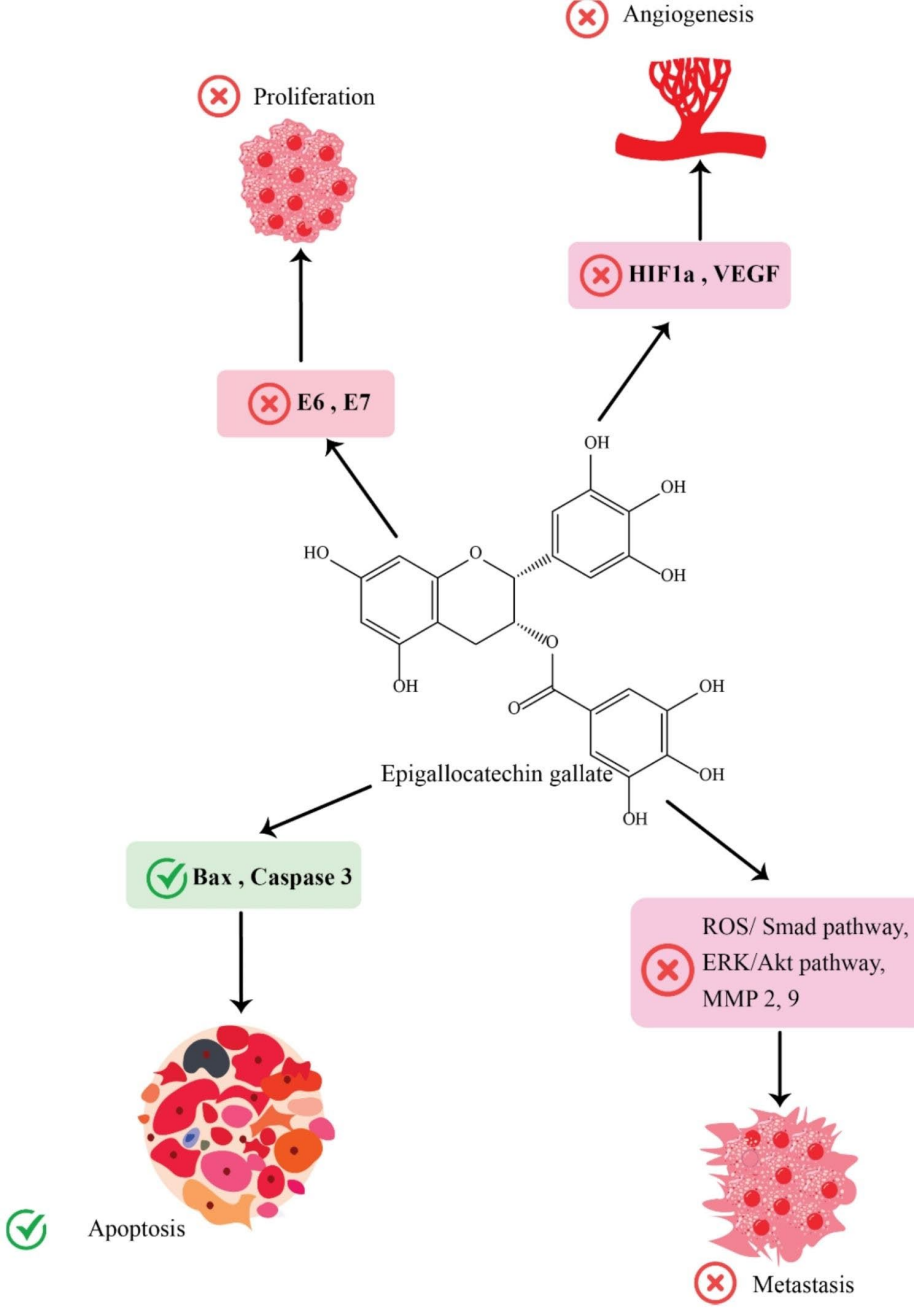
The role of green tea and its derivatives in cervical cancer

S, G1, G2, and M are the four phases of mitosis. Polyphenols E and epigallocatechin gallate both suppressed the growth of immortalized cervical cancer and epithelial cells. A dose-dependent relationship between apoptosis induction as well as changes to the cell cycle was discovered. EGCG-induced mitotic stoppage in squamous cervical cancer at low concentrations (0–25 µg/mL) Cells Me180 [27]. In many different cell lines and at different the cell cycle stages, EGCG was also demonstrated to prevent cell division. In HeLa cells, theaflavins and EGCG suppressed proliferation by inducing sub-G1 phase arrest in the dose- and time-dependent manner, respectively [62]. EGCG promoted G1 phase arrest and altered associated gene expression in HPV16-related CaSki cells [36]. In G2/M stages, in SiHa and HeLa cell lines, EGCG may suppress cell growth through a dose- and time-dependent way, respectively. Tumor suppressors, such as the cyclin-dependent kinase inhibitors (CKI) and P53 protein are secreted by organisms to interrupt the cell cycle of tumor cells [63]. Furthermore, EGCG boosted P53 expression in a dose-dependent way. Moreover, it has been indicated that Src homolog-2 domain transforming protein binds to the insulin-like growth factor receptor (IGF-1R), a tyrosine kinase receptor, to promote mitosis, anti-apoptosis and transformation in cancer cells [64]. For cancer cells to multiply, the extracellular signal-regulated protein kinases ERK1/2 and the epidermal growth factor receptor (EGFR) must both be activated. Via raising the levels of CKIs and P53 (P27/KIP-1, P21/WAF-1), EGCG inactivated ERK1/2 protein kinases and EGFR, leading to G1 arrest and enhanced cell death in multiple cervical cancer cell lines [65]. The polo-box domain (PBD), which is found in green tea catechin, has been shown to be a potent PBD inhibitor in PLK1 via inhibiting phosphopeptide’s binding to the PBD’s C-terminal. Additionally, By interfering with PLK1’s proper cellular localisation, EGC, a distinct kind of catechin, has the capability to lead to cell-cycle arrest within G2/M as well as S phases, ultimately inducing death in a number of cancer cells, including HeLa cells [66]. EGCG efficiently inhibited the ERKs’ phosphorylation through a dose-dependent way, while also reducing IGF-1R activity and its capacity to connect with the insulin-like growth factor (IGF-1), which prevented HeLa cells from proliferating and undergoing anchorage-independent transformation [67]. Potentially, P21 that is the inhibitor of cyclin/cyclin-dependent kinase complexes, was produced more often as a result of EGCG, resulting in mitotic arrest, which caused G2 arrest [37]. In addition, Polo-like kinase 1 (PLK1), which has been recognized among the possible therapeutic targets for cancer treatment, is essential for mitotic progression and cell-cycle regulation [68, 69] (Fig. 1).

## In vitro **studies about grean tea and EGCG, and cervical cancer**

Studies conducted in vitro have looked at how EGCG and green tea affect cervical cancer therapy. In a research, the impacts of green tea’s epigallocatechin gallate as well as polyphenol E upon immortalized cervical cancer cells and epithelial cells were assessed. Epigallocatechin gallate and polyphenol E inhibited immortalized cancer cell growth and cervical epithelial. Apoptosis activation along with changes to the cell cycle have been seen in a dose-dependent way. Green tea constituents inhibited the expression of the HPV-E7 protein [27]. Additionally, the mechanism of tea polyphenols’ antiproliferative and apoptotic effects on HeLa cervical cancer cells that are positive for the hpv-18 has been evaluated. After 24 h, treating HeLa cells with EGCG and polyphenol theaflavins (TF) from black tea clearly inhibited proliferation in a time- as well as a concentration-dependent way while dose-dependently bringing about the sub-G1 phase. Reactive oxygen species production, cytochrome c release, expression of p53, the Bax/Bcl-2 ratio, procaspase-3 and − 9, poly(ADP-ribose)-polymerase, and poly(ADP-ribose)-polymerase cleavage all increased with a decrease in potential of mitochondrial membrane, deonstrating the involvement of a mitochondria-related mechanism. Additionally, TF and EGCG reduced the activity of cyclooxygenase-2 by inhibiting the nuclear factor-kappaB (NF-kappaB) and Akt activation via inhibiting kappaBalpha, kappaBbeta subunit inhibitor phosphorylation along with subsequent degradation. Additionally, The amount of cyclin D1, an NF-kappaB transcriptional target, was much lower [62].

In a different research, CaSki cells which are a cell line of cervical cancer associated with the human papillomavirus (HPV)-16, were used to test the anticancer properties of EGCG. The growth-inhibitory effects of EGCG in CaSki cells were dose-dependent, with the inhibitory dosage (ID) [50] of around 35 microM. Apoptotic cells have been observable after 24 h at 100 microM EGCG once CaSki cells have been examined more in EGCG-induced apoptosis. At 35 microM EGCG, however, apoptotic cells’ negligible increase has been seen. Cell cycles in the G1 stage have been stopped been at 35 microM EGCG, indicating that cell cycle arrests may take place before apoptosis though. Using a 384 cDNA microarray to measure the gene expression of CaSki cells, it was shown that EGCG administration altered the gene expression. Over time, EGCG more than doubled the downregulation of 16 genes’ expression. Contrarily, Four genes’ expression levels were more than doubled by EGCG, indicating a potential function for EGCG in gene regulation [36]. According to a different research, EGCG may be useful for both cervical cancer prevention and therapy [48]. Additionally, the EGCG impact upon proliferative capability of HeLa cells has been examined using a cell viability experiment, and the outcomes were verified using nuclear morphological analysis, a cell cycle analysis as well as a DNA laddering assay. Outcomes demonstrated that Growth inhibition by EGCG was dose- and time-dependent in HeLa cells. It was discovered that EGCG-mediated cell death occurred via apoptosis. Surprisingly, EGCG substantially suppressed HeLa cell invasion and migration while also modulating associated genes’ expression (TIMP-1 and MMP-9) [34].

Sah et al. investigated the bioactive green tea polyphenol epigallocatechin-3-gallate (EGCG) [65] for its impact on cervical cells’ EGFR signaling. The epidermal growth factor-dependent mitogen-activated protein kinases EGFR as well as ERK1/2 are prevented from being activated by EGCG. Additionally, EGCG inhibits EGFR-dependent AKT activation. The EGCG-dependent decline in AKT and ERK activity is associated with decreased downstream substrates’ phosphorylation such as p90RSK, BAD as well as FKHR. Increased p27(KIP-1), p53, and p21(WAF-1) levels, decreased cyclin E levels, as well as decreased CDK2 kinase activity are all connected to these alterations. In accordance with our findings, TUNEL (terminal deoxynucleotidyl-transferase-mediated dUTP nick end labeling) and flow cytometry staining demonstrated EGCG-dependent G [1] arrest. Additionally, prolonged EGCG administration led to apoptotic cell death. Cell-free experiments showed that EGCG inhibits AKT and ERK1/2 directly in addition to EGFR, indicating that it suppresses EGF-dependent signaling at numerous levels concurrently. It’s significant that Since EGCG has no impact on the activation of JNK, which is EGFR-dependent, the inhibition of EGCG is selective [65].

Additionally, In human hepatoma (HepG2) and cervical cancer (HeLa) cells, the impacts of EGCG and green tea extract upon production of VEGF and HIF-1alpha were assessed. The outcomes demonstrated that, EGCG and Green tea extract substantially decreased serum- and hypoxia-induced HIF-1alpha protein accumulation in these cancer cells, but no impact has been seen on the expression of HIF-1alpha mRNA. EGCG and green tea extract also substantially decreased expression of VEGF via inhibiting mRNA and protein levels of HIF-1alpha protein. By inhibiting extracellular signal-regulated kinase 1/2 signaling pathways, the phosphatidylinositol 3-kinase/Akt and promoting HIF-1alpha protein degradation via the proteasome system, EGCG and green tea extract appear to reduce hypoxia-induced HIF-1alpha protein buildup. Furthermore, By inhibiting the phosphatidylinositol 3-kinase/Akt/mammalian target of rapamycin signaling pathways that are substantial in the protein translational machinery cascade, EGCG and green tea extract reduced serum-induced HIF-1alpha protein as well as the expression of VEGF [50]. In another study, Green tea extract (0-250 g/ml) and EGCG (0-100 mol/L) both inhibited cancer cell survival in a dose-dependent way. Furthermore, pre-incubation with EGCG (60 mol/L) as well as green tea extract (80 g/ml) dramatically reversed TGF-β effects in SiHa and Hela cells via reducing Vimentin, Slug, ZEB, Snail, and Twist expression along with raising expression of E-cadherin. EGCG and Green tea extract’s molecular mechanism for inhibiting TGF-induced EMT disrupted Smad signaling and ROS generation. EGCG and green tea extract may drastically reduce levels of ROS, Smad2/3 phosphorylation, DNA binding, translocation, and Smad activity in TGF-1β treated cervical cancer cell lines [42].

## In vivo **studies about grean tea and EGCG, and cervical cancer**

The impact of dietary supplementation with a nutritional mixture (NM) including proline, green tea extract, lysine ascorbic acid, and different micronutrients upon expression of extracellular matrix (ECM) proteins within HeLa cell xenografts was examined via an animal research [70]. The study’s results showed that NM significantly suppressed (by 59%) the development of HeLa xenografts in naked mice. When compared to control animals’ tumors, those from the NM group expressed hardly any collagen Internally or inside the fibrous capsule, I. Cancers in control group had numerous nucleated cells and diffuse capsular as well as cytoplasmic collagen IV. The NM treatment significantly boosted collagen IV synthesis and caused the formation of a thick fibrous collagen IV network with chambers around living nucleated cells and a significant quantity of necrotic cell debris. In contrast to tumors of control group, which had lower total fibronectin with sporadic internal staining as well as less in the fibrous capsule, NM-fed mice tumors displayed a vlearly defined boundary of fibronectin in the capsule as well as strong patches of staining internally. The tumors of control group displayed inner areas of elevated PAS staining, but those from the NM-treated group’s had a more uniform diffuse pattern of PAS staining [70]. In addition, in another study, these reasechers showed that Ki67, matrix metalloproteinase (MMP)-9 and − 2, terminal deoxynucleotidyl transferase dUTP nick end labeling, B-cell lymphoma 2, vascular endothelial growth factor, cyclooxygenase 2 (COX-2), S-transferase π and inducible nitric oxide synthase all showed that staining in the NM group was less intense and occurred less frequently than in the control group [71]. Furthermore, EGCG (25 M) boosted the efficiency of cisplatin therapy in HeLa cells via modulating the NF-κB, COX-2, p65, p-mTOR and p-Akt pathways, while increasing Nrf2/HO-1 expression levels in combination therapy [29].

Siddiqui et al. [49] found that EGCG at 5 and 10 g/ml doses enhanced the levels of caspase-3 in human cells. When compared to non-EGCG controls, cytosmears as well as slices of cervical cancer tissue cultivated with EGCG demonstrated improved differentiation and an raised amount of apoptotic cells. In 48 h, Those cells rose in number greater than in a 24-hour period. EGCG at 5 and 10 µg/ml doses increased apoptotic readiness in human cells as well as produced apoptotic alteration in cervical cancer cells [49]. In another trial, when given green tea extract, the response rate was 69% (35/51) as opposed to 10% (4/39) for the untreated controls [72]. Furthermore, combining EGCG (50–100 µM) with retinoic acid (RA) (1 µM) boosted the antiproliferative impact in adenocarcinoma cell lines, while either RA or EGCG therapy individually resulted in a lower responsive reaction in these cells. Neither apoptosis nor telomerase activity were influenced by EGCG or RA by itself. Combining EGCG with RA led to apoptosis and decreased telomerase activity in adenocarcinoma cell lines. These findings corroborated previous findings of an antiproliferative impact of RA and/or EGCG in cervical cancer cells [47]. Furthermore, the mixture of tea polyphenols (25–125 µg/mL) with bleomycin (5–25 µM/mL) therapy triggered apoptosis via caspase-9, caspase-8, caspase-3 activation, p53 overexpression, and Bcl-2 upregulation [73]. Doxorubicin and EGCG together boosted autophagic flow and sped up autophagosome formation. Furthermore, the combination of photothermal and chemotherapy has the potential to significantly increase anticancer effectiveness by ablating the tumor [58].

## Human studies on grean tea and EGCG, in cervical cancer

The laboratory experiment presented conclusive proof that EGCG is able to influence multiple molecular targets and hinder the development of cancer by preventing its initiation, promotion and progression. However, in order to determine the true effectiveness of EGCG in treating cancer, further studies using human participants are necessary. Various clinical trials have already confirmed the potential of EGCG in preventing cancer in humans. In order to assess the effectiveness and safety of a tea drink containing EGCG in treating advanced serous or endometrioid ovarian cancer, a phase II study with a single arm and two stages was conducted. Women with stage III-IV ovarian cancer were recruited for the study and were required to consume 500 mL of double-brewed green tea every day until their cancer recurred or for 18 months of follow-up. The main goal of the study was to determine if there was a lack of recurrence after 18 months. In the first stage of the study, 16 women were evaluated and only 5 of them did not experience a recurrence after 18 months following complete response. As a result, the clinical trial was stopped. The participants generally followed the instructions and consumed the double-brewed green tea regularly, but six women did not complete the 18-month follow-up. No severe adverse effects were observed, however it seems that taking double-brewed green tea does not effectively prevent recurrence in women with advanced stage ovarian cancer after receiving standard treatment [74].

A study was conducted to examine the tolerability, safety, and effectiveness of using topical EGCG as a treatment for radiation dermatitis in breast cancer patients undergoing adjuvant radiotherapy. The participants were breast cancer patients who had received radiotherapy to their chest wall after mastectomy. During the first week of radiation dermatitis, the green tea compound was sprayed onto the affected area and was continued for two weeks after the end of radiotherapy. The concentration of EGCG was gradually increased from 40 to 660 micromol per liter in 7 levels, with 3 to 6 patients in each level. Only one patient experienced skin redness, which was found to be associated with the treatment at a concentration of 140 micromol per liter. However, other patients who received this concentration did not experience any toxicity. No other acute toxicities were reported in relation to EGCG. Eight patients developed Grade 2 radiation dermatitis during or after radiotherapy. The use of topical EGCG was well tolerated and no maximum tolerated dose was identified [75].

Various scientific studies conducted with a randomized control group have presented inconsistent findings regarding the effectiveness of green tea extract in preventing or treating cervical lesions. One specific study, which utilized a randomized phase II trial method, looked into the impact of a specific green tea extract, called Polyphenon E, on patients infected with HPV and displays of low-grade cervical intraepithelial neoplasia (CIN). This particular trial did not establish any link between the use of green tea extract and the prevention of cervical cancer due to the absence of infection clearance or the regression of lesions (registered under NCT00303823). However, another study that investigated the use of Polyphenol E and EGCG, through ointment or 200 mg capsules, in women with HPV infected cervical lesions, did demonstrate a successful response in treating such lesions [72].

## Pharmacokinetic, bioavailability and metabolism of EGCG

It is crucial to have a thorough understanding of the pharmacokinetics and physiological impacts of EGCG. By utilizing Lipinski’s rule of 5, we can anticipate the oral bioavailability of this catechin [76]. Although EGCG has a logP value of less than 5, it does have eight hydroxyl groups which act as hydrogen bond donors, surpassing the limit of 5. Additionally, with a molecular weight of 458.372 g/mol, it is near 500, which violates two criteria. Due to this, it is expected that EGCG will have low bioavailability when taken orally [77].

A preclinical research conducted by Chen et al. revealed that EGCG has limited effectiveness in terms of absorption (with low Cmax value) and is primarily eliminated through bile (as evidenced by high AUC values in the intestine) [78]. The research findings show that when given through an IV, EGCG tends to spread throughout the outer areas of the body (with K12 being greater than K21). Additionally, EGCG has a long duration of presence in the body and is eliminated slowly (with a long t1/2 and low CL). However, there may be greater disparities in how it is broken down in humans compared to the rat model due to differences between the species. This is due to EGCG’s physical and chemical characteristics that determine its rate of absorption, metabolism, and excretion from the body. It falls into the BCS class III category of drugs, meaning it has low oral bioavailability due to its hydrophilic nature [76].

After being digested, green tea catechins have a very low stability and only 20% of them remain intact. Out of those, EGCG is the most sensitive and less than 10% of it can be absorbed [79]. EGCG, a type of tea catechin, has the least potential for therapeutic benefits due to its limited bioavailability, which is determined by factors such as metabolism and stability. When taken orally, EGCG undergoes metabolic modifications, starting in the mouth with the hydrolysis and degalloylation by salivary enzymes called catechin esterases [80]. The process of biotransformation occurs within the liver for EGCG, where it is converted through glucuronidation by UDP-glucuronosyltransferase (UGT), sulfation by sulfotransferase (SULT) and methylation by catechol-O-methyltransferase (COMT) [81]. The digestive microbes in the gut play a significant role in the breakdown of EGCG. Both laboratory and animal studies have revealed that these gut bacteria are capable of breaking down and reducing the effectiveness of EGCG. A study using the cecum of a pig revealed that all of the EGCG was transformed by the gut bacteria within a short time frame of 4 to 8 h [82]. Moreover, the amount of EGCG and its metabolites within the cell is regulated by ATP-dependent multidrug resistance-associated protein (MRP) efflux pumps. These pumps, specifically MRP1 and MRP2, transport EGCG out of the cell and into the bloodstream or intestinal space, thereby increasing their availability within the body. MRP2 also plays a role in secreting absorbed EGCG from the intestine back into the intestinal lumen. However, if EGCG is not released into the intestinal lumen by the enterocyte, it may be absorbed into the portal circulation, leading to a decrease in bioavailability due to MRP2’s efflux. Additionally, the levels of MRP2 transcripts in the human jejunum are significantly higher compared to MRP1, suggesting that MRP2’s efflux of these molecules may be a major factor in limiting EGCG’s effectiveness in vivo [83].

The natural lack of stability of EGCG poses a challenge in its ability to be absorbed and have a meaningful impact. When applying results from laboratory tests to real-life scenarios, it is important to take into account the low absorption rate of this specific polyphenol. Because of the disparity between its performance in controlled experiments and real-life settings, it is challenging to provide concrete dosage recommendations for EGCG [84]. It is imperative to comprehend the elements that influence its ability to be utilized in order to address the issue and improve its effectiveness.

## Improvement of bioavailability of EGCG: nanotechnology comes into view

Nanotechnology is a multidisciplinary subject incorporating elements of biology, engineering, chemistry, and medicine. It utilizes nanosystems, which are artificially created tools with a size between 1 and 100 nanometers [85, 86]. The use of nanotechnology is currently under examination and being put into practice for detecting and treating cancer, through the creation of nanosensors and nanovectors [85]. Nanovectors refer to a type of tiny particles, known as nanoparticles (NPs), which are capable of carrying drugs or imaging substances with the purpose of delivering them to specific tumor cells in the body [85]. Numerous types of nanoparticles can be utilized for creating drug delivery systems that combat cancer, such as liposomes, magnetic NPs, and polymeric NPs, to name a few [85]. The potential for nanoparticles to act as effective carriers for delivering cancer-fighting drugs is vast, as they enhance the drug’s absorption, ability to dissolve, and availability for use in the body. Additionally, they shield the drug from breaking down too early and prolong its time in the bloodstream [85, 86]. Furthermore, NPs have the ability to boost drug concentration specifically in tumor cells by taking advantage of the enhanced permeability and retention effect (EPR). They also aid in penetrating cellular barriers and can be modified with surface functionalities to improve targeting specificity, ultimately reducing the potential toxic effects of drugs [87]. In addition, they allow for the drug to be taken orally, which is the most favored method of delivery for patients due to its ease and compliance [88].

Chemoprevention shows potential as a method for preventing, slowing down, or reversing cancer through the use of both natural and man-made substances, including EGCG. However, its effectiveness is currently hindered by both toxicity and inadequate delivery and absorption into the body [89]. To address these drawbacks, Siddiqui and colleagues proposed the idea of nanochemoprevention, which involves utilizing nanotechnology to enhance the effects and applicability of chemopreventive agents for cancer treatment [89]. Additionally, besides its uses in preventing cancer, EGCG may also play a significant part in enhancing the effects of chemotherapy. It has been demonstrated that EGCG can work together with well-known cancer-fighting drugs like doxorubicin, tamoxifen and paclitaxel in various types of cells [90]. Numerous investigations published in scholarly articles have utilized techniques involving nanotechnology, utilizing diverse sets of tiny particles to transport EGCG in order to specifically treat various forms of cancer in laboratory settings and living organisms. These findings will now be further elaborated, organized based on the specific type of nanoparticle employed. Gold NPs possess distinct physical and chemical characteristics, including their diminutive size, ability to exhibit plasmon resonance, capability to bind with amine and thiol molecules, significant atomic number, and compatibility with biological systems [91]. The typical procedure for creating these NPs consists of reducing Au (III) compounds, such as Chloroauric acid (HAuCl4) [91]. In general, a liquid mixture containing HAuCl4 is combined with another liquid containing a substance that causes reduction, resulting in the reduction of Au3 + and the resulting creation of gold NPs [91]. According to Nune et al., polyphenols have been found to serve as both reducing and capping agents in this process. This technique eliminates the need for an extra synthetic chemical, thereby promoting a more environmentally friendly approach known as “green chemistry” [92].

The researchers, Sanna et al., created gold nanoparticles through a method that closely resembled the one outlined by Nune et al. [92]. In simulated biological fluids, it was observed that gold nanoparticles bound with EGCG remained remarkably stable and retained the antioxidant properties of EGCG [93]. Furthermore, the study found that the nanoparticles effectively triggered the programmed cell death process, known as apoptosis, by activating caspase-3 in neuroblastoma SH-SY5Y-CFP-DEVD-YFP cells. This effect was observed to be dependent on the concentration of the nanoparticles and occurred after 72 h of exposure. The researchers also determined that the effectiveness of EGCG remained intact when attached to the surface of gold NPs [93].

Gelatin is widely utilized in both food and medical items, serving as a non-toxic polymer that can naturally break down over time [94]. It is distinguished by its mechanical, thermal, and swelling characteristics, which are greatly influenced by the level of crosslinking [94]. The team of Shutava and colleagues created nanoparticles using gelatin as a base material, and also tested their effectiveness with a covering of polystyrene sulfonate/polyallylamine hydrochloride obtained through the layer-by-layer method [95]. Gelatin NPs exhibited a prolonged release of EGCG, in contrast to non-coated NPs. The encapsulated form of EGCG retained its potency, effectively inhibiting the hepatocyte growth factor (HGF) and the resulting activation of cell signaling pathways that contribute to cell invasion in MDA-MD-23 breast cancer cells.

Liposomes are tiny spherical structures composed of a double layer of phospholipids that surrounds a watery inner space [96]. The presence of these structural characteristics allows for the containment of both fat-soluble and water-soluble medications [96]. Moreover, liposomes are able to be broken down by natural processes and do not display significant levels of harmful effects [96]. According to de Pace et al., they incorporated EGCG into the aqueous center of nanoliposomes composed of cholesterol and phosphatidylcholine, with a 0.2% chitosan coating [87]. The experiments conducted in a laboratory setting showed that the nanoparticles (NPs) greatly improved the stability of EGCG and prevented it from breaking down too quickly in both PBS and cell culture mediums. This was in stark contrast to free EGCG, which degraded at a much faster rate. Nanoencapsulation also resulted in a slower release of EGCG and a higher concentration of it in MCF7 breast cancer cells compared to free EGCG. Furthermore, treating the cells with both chitosan-coated and non-coated nanoliposomes resulted in different levels of EGCG in the cells, indicating that chitosan may increase cell absorption. When a dose of 10 mM of chitosan-coated liposomes was used, significant anti-proliferative and pro-apoptotic effects were observed, with a 40% decrease in MCF7 cell proliferation compared to native EGCG and a 27% induction of apoptosis in MCF7 cells [87]. Table 2 shows various nano-based platforms that are used EGCG for trating various cancers.

**Table 2:** Various nano-based platforms plus EGCG in different cancers

Type of cancer	Type of nano-formulation	Size	Admintsation route	Result (s)	Ref
Breast cancer	Gelatin NPs	200	-	Inhibit HGF in MDA-MD-231 breast cancer cell line	[95]
Prostate cancer	Chitosan NPs	150–200	-	Inhibit the tumor cell growth and proliferation	[88]
Colon cancer	PLGA NPs	127	-	Increase in DNA damage levels of oxaliplatin	[97]
Prostate cancer	PLA-PEG NPs	260	Intra-tumoral	Induction of apoptosis in prostate cancer PC3 cell line; inhibition of angiogenesis Significant decrease in tumor size in prostate cancer xenograft model	[89]
Bladder cancer	Gold NPs	20–1200	Oral Intra-tumoral or intra-peritoneal	Reduction in tumor volume	[98]
Neuroblastoma	Gold NPs	25.55	-	Induction of apoptosis	[93]
Basal cell carcinoma	Liposomes	157.4, 268.9	Topic and intra-tumoral	Great amount of EGCG deposition in tumor tissues in BCC model in female nude mice	[99]
Brast cancer	Chitosan-coated liposomes	16.4	-	Inhibit the tuomor cell proliferation and induce apoptosis in tumor cells	[87]
Prostate cancer	Ca/Al-NO3 LDH	-	-	Improve anti-tumor activites	[100]
Prostate cancer	Maltodextrin-gum arabic	120		Improve anti-tumor activites	[101]
Liver cancer	Ruthenium	73.59	-	Improve anti-tumor activites	[102]

## Green tea and epigenetics modification

Different species’ biological activities and processes depend heavily on a complex interplay between fundamental cellular systems that have been calibrated by genetic make-up and a complex web of cellular RNA expression patterns controlled by epigenetic regulation. The word “epigenetics” describes the variation in gene expression that occurs without any underlying changes to the genetic sequence itself [52]. The concept of epigenetics includes planned developmental changes as well as the genome’s capacity to recognize, transmit, and perpetuate environmental stimuli [53]. Conrad Waddington created the word “epigenetics” in 1942 to describe parts of development for which there was no mechanistic understanding at the time. He described it as “changes in phenotype without changes in genotype.“ The first epigenetic signature to be conclusively linked to the development of cancer was DNA methylation [54]. Biological variety, such as phenotypic variance among genetically identical people, is also fundamentally influenced by epigenetics [55]. In fact, in Apis mellifera species, epigenetic mechanisms fully explain the differences between worker bees and queen bees [56]. DNA methylation, histone changes, as well as microRNA gene silencing are 3 main elements of epigenetics [57]. In addition to the DNA template, epigenetic processes function to maintain gene expression patterns and hence channelize cell-type identities. Numerous studies on the enzymatic characterization of various chromatin states that promote or repress gene activity have long acknowledged the significance of this epigenetic regulation. It is now clear thanks to recent developments in science and technology that altered epigenetic regulation of gene expression has a substantial part in various disorders, including cancer. Contrary to the DNA sequence, which remains mostly constant throughout life, epigenetic patterns differ from one tissue to another, change with aging, and are susceptible to environmental exposures. Since epigenetic processes exist at the nexus of the environment and coordinated transcriptional regulation, it is this tendency for change that has drawn so much attention [58]. The epigenetic modifications’ part in the onset of cancer and other illnesses is now more understood. However, epigenetic states may also be disturbed by environmental factors or as we age. Epigenetic changes are being increasingly targeted as potential therapeutic targets for various types of cancers because of their reversible nature [59–62]. These epigenetic modifications show as regional changes in the gene promoters of several tumor-suppressor genes (TSGs) as well as worldwide changes in nucleosome packing that affect the gene transcription throughout neoplastic progression and initiation. HDAC and DNMT inhibitors are two epigenetic targeting strategies that are currently being researched. However, some drawbacks that now limit the widespread use of these synthetic epigenetic medicines include their lack of specificity, brief action times, and potential to alter healthy cells’ structural and functional patterns with unexpected results [63, 64]. Growing data over the past ten years has shown previously unheard-of hints that nutrition as well as environmental variables have a straightforward effect upon human epigenetic systems. It has been demonstrated that dietary polyphenols from green tea, soybeans, turmeric, broccoli, as well as other foods have a variety of cell regulatory functions in cancer cells. In recent times, it was demonstrated that different dietary polyphenols might partially exert their chemopreventive influences via modulating epigenetic machinery’s different elements. As a result, medications that target these changes are currently being researched in different human cancer patients [65, 66]. The most prevalent chemical constituents in green tea, catechins, including (-)-epigallocatechin-3-gallate (EGCG), were indicated to have antioxidant, anti-inflammatory, antiproliferative, antimetastatic, and anti-angiogenic effects, induce apoptosis or differentiation, inhibit telomerase activity, arrest the cell cycle as well as prevent DNA adduct formation [67–69].

## Epigallocatechin-3-gallate and DNA methylation

In a research, Ali Khan et al. examined how EGCG affected the epigenetic state of HeLa cells [70]. Inhibition experiments for histone deacetylase (HDAC) and DNA methyltransferase (DNMT) have been carried out, and RT-PCR and enzymatic activity assays, respectively, were used to measure HDAC1 and DNMT3B transcription levels. In addition, they used molecular modeling to investigate how EGCG contacts with HDAC1 and DNMT3B, as well as MS-PCR and RT-PCR to examine the promoter DNA methylation along with the expression of the retinoic acid receptor (RAR), death-associated protein kinase-1 (DAPK1) and cadherin 1 (CDH1) in EGCG-treated HeLa cells. The enzymatic activity of HDAC as well as DNMT has been shown to be substantially lowered in the current study’s time-dependent EGCG-treated HeLa cells. While there wasn’t amy discernible change in HDAC1 expression, DNMT3B’s expression was dramatically reduced with time. The suppression of HDAC1 and DNMT3B activity caused via EGCG has been confirmed more through molecular modeling information. Also, time-dependent exposure to EGCG caused discernible methylation alterations in promoter regions of known tumor-suppressor genes (TSGs), which led to their reactivation in HeLa cells. Overall, the results of the current investigation imply that EGCG could significantly influence the creation of innovative epigenetic-based therapies [70].

In HeLa [71] cervical cancer cells, Sundaram et al. investigated the impact of epigallocatechin gallate (EGCG) on signaling pathways, tumour suppressor genes and epigenetic modulators. histone modifiers (DNMT1, DNMT3A, DNMT3B, AURKC, AURKA, AURKB, KDM5C, KDM4A, PRMT6, PRMT7, HDAC5, UBE2B, HDAC7, HDAC6, and HDAC11) and DNA methyltransferases were among the epigenetic modifiers whose transcription was modulated by qRT-PCR. Additionally, EGCG was found to reduce the activity of DNA methyltransferases, histone methyltransferases (H3K9) and histone deacetylases in ELISA-based experiments. According to the results of molecular docking, DNMT3A, DNMT1, HDAC2, HDAC4, HDAC3, HDAC7, as well as EZH2 are only a few of the epigenetic enzymes that may be competitively inhibited by EGCG. The promoter hypermethylation’s reversal of tumor suppressor genes via transcriptional re-expression and quantitative methylation array of tumour suppressor genes such as PTEN, TP73, SOCS1, RAR, CDH1 and DAPK1 via qRT-PCR allowed for the inference of a functional result of these epigenetic changes. Downregulation of pro-inflammatory molecules such TERT, CCNB1, CCNB2, MMP2, and MMP7 as well as important signaling molecules of the Wnt, MAPK and PI3K pathways, metastasis regulators and cell cycle regulators. Also noted were IL6, PIK3C2B, PIK3CA, MAPK8, and PIK3C2B. KEGG analysis and in silico protein-protein interaction network analysis were used so as to identify active contribution of gene sets to cancer pathways. According to this research, anti-cancer mechanism of EGCG is explained in detail by the coordinated transcriptional change of multiple molecular targets along several signaling pathways as well as the reversal of tumor suppressor gene suppression via manipulation of epigenetic enzymes [71].

## Epigallocatechin-3-gallate and MicroRNA

MicroRNAs (miRNAs/miRs), also known as small non-coding RNA molecules control the expression of protein-coding target genes in eukaryotes [72]. According to reports, miRNAs have a significant regulatory part in a number of vital biological processes like cell growth, migration, invasion, and metabolism [73]. Epigenetic changes, however, also have a substantial part in the development of cancer as well as its dissemination, according to some studies. Non-coding RNAs, such as miRNAs and long non-coding RNAs, were the subject of an increasing number of studies [74, 75]. MiRNAs may be applied in the therapy of different cancer types since they may be employed as biomarkers to evaluate disease features or progression [12]. Furthermore, abnormal histone modification and DNA methylation in cervical cancer received attention and were the subject of in-depth research. Research has focused more and more on non-coding RNAs, especially long non-coding RNAs and miRNAs. MiRNAs may be applied in the therapy of different cancer types since they may be employed as biomarkers to evaluate disease features or progression [76]. Cervical malignancy has been linked to genetic mutation, which has been acknowledged [77]. Numerous studies have recently concentrated on analyzing the molecular underpinnings and processes of epigenetic alterations in cervical cancer like histone modification and the function of DNA methylation as well as the prognostic, therapeutic and diagnostic potentials of miRNAs [78, 79].

According to earlier research, some miRNAs are downregulated whereas others are increased in cervical cancer [80, 81]. MiR-203 expression has been shown to be downregulated in tumor tissue as well as cervical cancer cells [81]. Our team recently found that miR-125b inhibits PI3K/Akt/mTOR activity to reduce tumor development, recommending that it might be a useful therapeutic target for cancer therapy [82]. Additionally, a microarray investigation showed that grade II–III HPV16 + cervical intraepithelial neoplasia tumors had miR-29a downregulation whereas miR-210 was increased in cervical cancer [83].

Zhu et al. looked into the inhibition of microRNA (miR) expression that the EGCG has on the development of cervical cancer cell lines that had been exposed to several high-risk human papillomavirus (HPV) subtypes [84]. The impacts of EGCG upon HeLa cell proliferation have been considerably dose- and time-dependent, with the IC50 values at 24 and 48 h being 90.74 and 72.74 g/ml, respectively. Using quantitative polymerase chain reaction analysis, the expression of miR-210, miR-203, miR-29a and miR-125b in HeLa (HPV16/18+), SiHa (HPV16+), CaSki (HPV16+), and also C33A (HPV-) cell lines has been determined. MiR-125b and MiR-203 have been dramatically downregulated in CA33 cells via EGCG, but miR-210 was significantly increased by EGCG at 40 and 60 µg/ml concentrations. By ≤ 80 g/ml of EGCG to HeLa cells, miR-125b was considerably downregulated, whereas miR-29 and miR-210 have been substantially increased. EGCG dramatically increased the levels of miR-210, miR-125b and miR-29a in CaSki cells. EGCG dramatically increased miR-125b and miR-203 levels in SiHa cells. The findings of the current research conclude that EGCG inhibits cervical carcinoma cells’ proliferation, presumably through modulating the expression of miRs, indicating that these compounds may be therapeutic targets for treating and preventing cervical cancer [84].

## Conclusions

In conclusion, Cervical cancer preventive and treatment benefits of green tea’s main bioactive component, EGCG, have been demonstrated in research. Studies conducted in vitro suggested that EGCG may have an impact on a number of intracellular signaling pathways and receptors implicated in the growth and survival of cancer. Although the outcomes of clinical trials have been conflicting, in vivo research further indicated EGCG’s advantages in preventing and shrinking of tumors. There is proof that green tea improves therapy of cervical cancer, even if EGCG medicines are still controversial. However, more investigation is necessary to come to firmer findings and bolder recommendations about the possible use of green tea in cancer treatment, either individually or in conjunction with chemotherapeutic medications. It is noteworthy that several of the EGCG-interacting proteins addressed in this research have not yet had their effects on cervical cancer completely defined. Given EGCG’s pro- and antioxidant actions, there are special potential for its usage in treating cancer that depend on the severity of the condition.

Our knowledge of the effects of nutrition and botanical dietary supplements on epigenetic processes will also significantly improve over the next ten years as a result of growing technology and falling costs of measuring changes in epigenetic alterations. We anticipate that future research will support a growing role for food in regulating DNA methylation and non-coding RNAs, even if the influence of dietary exposure on epigenetics is mild. This is because dietary exposures are frequent and long-term in nature. Studies conducted on in vitro cell cultures clearly show that prolonged GT therapy can alter epigenetic alterations and restart gene expression.

This review outlines several of the potential physiological effects of EGCG and offers suggestions for further studies. It is crucial to keep researching EGCG as a potential medicinal agent due to its widespread accessibility and availability as a natural substance.

## Data Availability

Not applicable.
